# Letter to the Editor: In Response to Sarkar *et al*.

**DOI:** 10.1002/acm2.13277

**Published:** 2021-05-27

**Authors:** Damodar Pokhrel, Justin Visak

**Affiliations:** ^1^ Department of Radiation Medicine Medical Physics Graduate program University of Kentucky Lexington KY USA

**Keywords:** Co‐planar geometry, Halcyon, Lung SBRT, VMAT

Dear Editor

We read with interest the letter to the editor drafted by Sarkar *et al*. in regards to our recently published paper: Pokhrel et al. Clinical validation of ring‐mounted halcyon Linac for lung SBRT: comparison to SBRT‐dedicated C‐arm Linac treatments. J Appl Clin Med Phys 2021; 22(1); 261‐270.

We appreciate the interest in our recently published paper by the author, however, after carefully reviewing this draft, we found several flaws in the letter to the editor. We strongly feel the assertions presented by Sakar *et al*. do not specifically pertain to our paper^1^ as the comparisons between our study and theirs are not appropriate to make. The author presents inconsistent and incomplete treatment planning information about their study that is highly misleading and will be ambiguous to both the radiation therapy community and fellow JACMP readers, if published. It is inadequate to draw any conclusions on the ability of the Halcyon Linac to meet RTOG‐0618 criteria^2^, ^3^ for lung SBRT based on the very limited information presented in the letter to the editor. We have several comments outlined below.

1. The author’s study immediately significantly deviates from our paper as they used a 6 MV flattened beam (much higher mean energy and corresponding PDD) with significantly different beam characteristics. Alternatively, we used the closest possible beam profile and characteristics via the 6MV‐FFF beam on C‐arm TrueBeam Linac for the validation of single‐energy 6MV‐FFF Halcyon for lung SBRT treatments. This would greatly affect the feasibility of meeting RTOG‐0618 requirements.^3^
2. In terms of the dosing prescription scheme, the author states their planning study was for 60 Gy in five fractions using RTOG‐0618 criteria. We believe that the most common prescription of the RTOG‐0618 trial is a 54 Gy in three fractions scheme.^3^ In our paper, we demonstrated that the coplanar Halcyon VMAT plans are similar to our clinical non‐coplanar (0°, ±10°, couch positions) TrueBeam VMAT plans and met RTOG‐0813 criteria for 50 Gy in five fractions for these SBRT treatments.^4^ This 10 Gy dose increase may solely explain why the two of the nine patients in their limited study did not meet the normal lung V20 Gy <15% requirement or spinal cord dose tolerance set‐forth in the (differing from ours) RTOG‐0618 criteria. Along with this, many other critical parameters were not included in the letter to the editor. All of which must be mentioned before it is appropriate to compare to our study. The author failed to provide any information about the following: tumor size and location, planning technique (3D‐static or dynamic conformal arcs on Novalis or VMAT?), similar planning objectives between Novalis or Halcyon plans (identical objectives deployed or not?) or the final dose calculation algorithm (e.g., AAA vs. AcurosXB vs. advanced Monte Carlo based calculations?). All of these will significantly affect their final plan quality and we think that this information is of utmost importance before any comparison may be made to our study. Along with incomplete planning information, the author additionally fails to acknowledge that the well‐understood inter‐planner variability and institutional experience will be a dictating factor in the final plan quality. Therefore, based on the inconsistent and incomplete information provided in the letter to the editor, we do not think we can definitely conclude that Halcyon Linac cannot meet RTOG‐0618 requirements. Detailed study merits future investigation on this ultra‐high dosing scheme.
3. In our study and at our institution, both lung SBRT plans were normalized to ensure 95% of the PTV is covered by 100% of the prescribed dose with a 120–130% hot spot in the GTV. This is accomplished by prescribing the dose to the tumor periphery with a sharp penumbra using the 70–80% isodose line. This prescription effectively constraints the hotspot to the center of the GTV and provides a sharp dose gradient for heterogenous SBRT dose distribution. Ultimately, this reduces the dose to the normal lung as intended in the RTOG protocols. In contrast, the author presents an example of a more homogenous dose distribution based on their reported normalization conditions. By creating a more homogenous dose distribution, normal lung dose will increase because of the inherent increase in intermediate dose spill that our institution avoids. To compound this normalization condition, the tumor size was not reported by the author for their two specific cases where a larger tumor size could explain the violation of RTOG‐0618 criteria observed.
4. To characterize the Novalis non‐coplanar lung SBRT capabilities with respect to the Halcyon, more information is required in the letter to the editor to not confuse any potential readers. The author mentioned that 40% NTX plans were non‐coplanar beams; however, they did not mention the technique deployed (e.g., 3D static fields or DCA or IMRT or VMAT arcs) nor the couch angles chosen for these static/arc geometry beams. We do acknowledge that due to collision issues there are limited angles available for non‐coplanar geometry on a C‐arm Linac for lung SBRT. However, its unwise to infer the angles used for their respective NTX plans despite the comment of a “similar” set‐up (line 11 of the author’s letter) to our study. It is understood that using an exotic non‐coplanar beams/arcs arrangement will yield high‐quality and RTOG compliant treatment plans. Unfortunately, this does not guarantee for the plan deliverability as it may render collision issues (especially in the VMAT delivery) or impractical longer treatment delivery times for select distressed pulmonary cancer patients. It is for these reasons more information about the non‐coplanar NTX plans must be included in the author’s letter as it cannot be certain uncommon beam geometry was deployed for these compliant plans. This is one of the reasons the author’s claim that the Halcyon Linac is uncapable of meeting RTOG‐0618 criteria for these 2 patients is clouded.
5. It is acknowledged by the author that the NTX flattened beam significantly differs from the Halcyon beam characteristics. This includes differences in mean energy, PDD, beam profile, and multi‐leaf collimators (MLC) characteristics. The Halcyon Linac is equipped with a next‐generation SX2 MLC with improved and differing values for MLC size (equivalent 5 mm width at isocenter), less leakage, and transmission dose of <0.5%. Therefore, we used the closest possible 6MV‐FFF beam energy of TrueBeam Linac (with standard 120 Millennium MLC) to compare our Halcyon validation plans for lung SBRT. We believe that this will better characterize the mechanical improvements or limitations of the Halcyon V2.0 for lung SBRT treatments. Without a detailed dosimetric study, we believe it is not appropriate to make any comparison with our paper as using an FFF‐beam could potentially increase the dose coverage at the tumor periphery and reduce the out‐of‐target normal tissue dose.^5^, ^6^ This is an important rationale to note as to why the letter to the editor does not specifically concern our paper, but rather would potentially serve as an interesting stand‐alone study to the greater medical physics community.
6. Halcyon gantry rotation speed is “4 RPM” not “2 RPM.”
7. We acknowledge and agree with the author that maximal dose rate differences between modalities may play a role in final beam‐shaping capabilities in the VMAT planning of these lung SBRT treatments. In our Halcyon lung SBRT validation study with the RTOG‐0813 protocol, we use the TrueBeam 6MV‐FFF beam with a maximal possible dose rate setting of 1400MU/min vs 6MV‐FFF on Halcyon with 800MU/min. Unfortunately, we cannot be certain the author deployed an inverse planning technique for their NTX plans as no specific mention of their planning technique was included in the letter to the editor.
8. In their example case, for the same prescription, the author reported almost 80% higher total MU with NTX compared to the Halcyon plan. We find this increase extremely concerning if the same technique is used between the modalities. This may indicate a fundamental issue with the study described in the letter to the editor. For the given prescription and similar planning technique, we believe that non‐coplanar geometry should give similar or even less total MU, in general, due to relatively less chance of beams or arcs overlapped in the target. Our experience for lung SBRT is that for the same prescription and identical target coverage, we get similar MU, similar intermediate dose‐spillage, but slightly higher low dose volume with 6MV‐FFF Halcyon compared to 6MV‐FFF TrueBeam. We reported this in our paper via slightly higher volumetric doses to the OAR such as dose to 10 cc of skin.^1^ Our slightly higher low dose volume is to be expected due to the beam characteristics of the lower mean energy (~1.3 MeV) of the 6MV‐FFF Halcyon beam. This substantial increase of MU reported on their NTX plan by the author must be further investigated and described before any assumption can be made on the Halcyon’s ability to meet RTOG‐0618 or comparison to our results that did not use RTOG‐0618 prescription.
9. We agree with the author that, unlike intracranial SRS, there is a limited scope of non‐coplanar geometry in the extracranial region due to the gantry collision issues. Clinics that use a advanced treatment planning system with VMAT or IMRT optimization may deploy additional approaches such as optimal values of normal tissue objectives, OAR avoidance feature or sectors avoidance (in VMAT) to further compensate for lack of full angular capabilities in these treatments and further spare the OAR. Author needs to clarify the details of their treatment planning approach.
10. In our paper, in addition to achieving the clinically acceptable and RTOG‐0813 compliant plan quality for select lung SBRT patients, we present a discussion about Halcyon delivery efficiency. Our concern with the “4π algorithm”^7^ or so‐called “limited 2π per the author of this letter” is that it used up to 30 fully optimized coplanar/non‐coplanar IMRT fields. Likely, delivering 30 c/n‐coplanar IMRT treatment fields to treat lung SBRT patients would be clinically unfeasible in the current clinical setting due to potential collision issues and the therapists’ need to enter the treatment room many times. This would greatly prolong treatment times, introducing patient discomfort and distress and potentially increasing the chance of intrafraction motion errors. Additionally, this would overall slow down the clinic efficiency. Utilizing Halcyon VMAT overcomes all these concerns as demonstrated in our paper.
11. We strongly disagree with the author’s unproven claim that the Halcyon planning technique is limited. Based on our clinical experience and the currently available literature,^8^, ^9^ the coplanar geometry Halcyon Linac is more than capable of providing a fast, safe, high‐quality, and convenient treatment including for the select prospective lung SBRT patients.^1^ As a reminder, the SX2 stacked and staggered MLC equipped on the Halcyon provides a 5 mm equivalent width at isocenter with ultra‐low leakage and transmission dose of MLC (<0.5%) that may contribute to final plan quality. Likewise, the more rounded MLC design offers a much sharper penumbra width as reflected with the small, ​~0.1 mm dosimetric leaf gap. When the improved MLC and increased gantry rotational capabilities of the Halcyon are fully considered, it is evident that the Halcyon could provide similar or better‐quality plans including a relatively faster treatment delivery for select lung SBRT patients as shown in our paper. Moreover, in the modern era of inverse planning, we believe that the planner’s skill, interplanar variability, and experience (among many other factors) are critical in the generation of high‐quality clinical plans. Therefore, it is not reasonable to grossly assert that the Halcyon planning technique is seriously limited without providing ample context in the author’s letter to the editor.
12. As the author suggested, a small yaw rotation to the couch and a small gantry tilt is similar to some advanced CT simulator, however, to incorporate this feature on Halcyon, we think that would need a major structural re‐design of the current Halcyon Linac by the Varian Medical Systems (Palo Alto, CA). Therefore, the author’s conclusion suggesting: “it will result in a better dosimetric outcome” is premature and yet to be demonstrated (or designed).

Therefore, we believe that the generation of a high‐quality clinical lung SBRT plan does not only depend on the coplanarity (or lack‐thereof) beam geometry on a case‐by‐case basis, but also depends upon the fine details of every aspect of the treatment planning approach and the patient selection that we described above. To conclude, based on the deficiencies as noted above, the author’s letter to the editor is not relevant or consistent with our published paper on the Halcyon Linac’s lung SBRT capabilities. This includes fundamental differences between our detailed study and the author’s very limited study in terms of concept, methodology, and results that we believe provide clinical value to the fellow Halcyon users. Additionally, due to the incomplete and inconsistent description of the author’s limited study, it may create confusion and ambiguity concerning the Halcyon’s ability (to the Halcyon users) to commission and implement the SBRT protocols in the clinic for the prospective patient’s treatment.

## Data Availability

Research data are not shared.
